# Supplementary material for: Evaluating the potential impact of rubella-containing vaccine introduction on congenital rubella syndrome in Afghanistan, Dem. Republic of Congo, Ethiopia, Nigeria, and Pakistan: A mathematical modeling study
Source: PLOS Glob Public Health. 2024 Jan 16;4(1):e0002656. doi: 10.1371/journal.pgph.0002656 (PMC10791005; doi:10.1371/journal.pgph.0002656)
Supplement: S1 Appendix — (DOCX) [file pgph.0002656.s002.docx]

## Compartmental SEIR model

The compartmental SEIR model used in this study was proposed in [1]. The parameters of the disease model are shown in Table A in S1 Appendix. We obtained from [1] the rate at which individuals leave the exposed state, the recovery rate, the vaccine efficacy, and the probability that a child of an infected mother develops CRS. The force of infection was obtained from several studies, which are displayed in Table B in S1 Appendix. For ETH, NGA and PAK, we obtained the force of infection from the recommended scenarios from [1], using point-estimates stratified for the younger (<= 13 years old) and older (> 13 years old) populations (see Table D in S1 Appendix). For AFG we obtained point-estimates based on the force of infection for the region as reported in [1] (see Table E in S1 Appendix). Lastly, for COD we used sub-national estimates from [2] to calculate a national-level estimate (see Table F in S1 Appendix). The model was implemented using Python 3.11 using NumPy as primary computation library.

**Fig A in S1 Appendix.** Flow diagram of compartmental disease model*.*


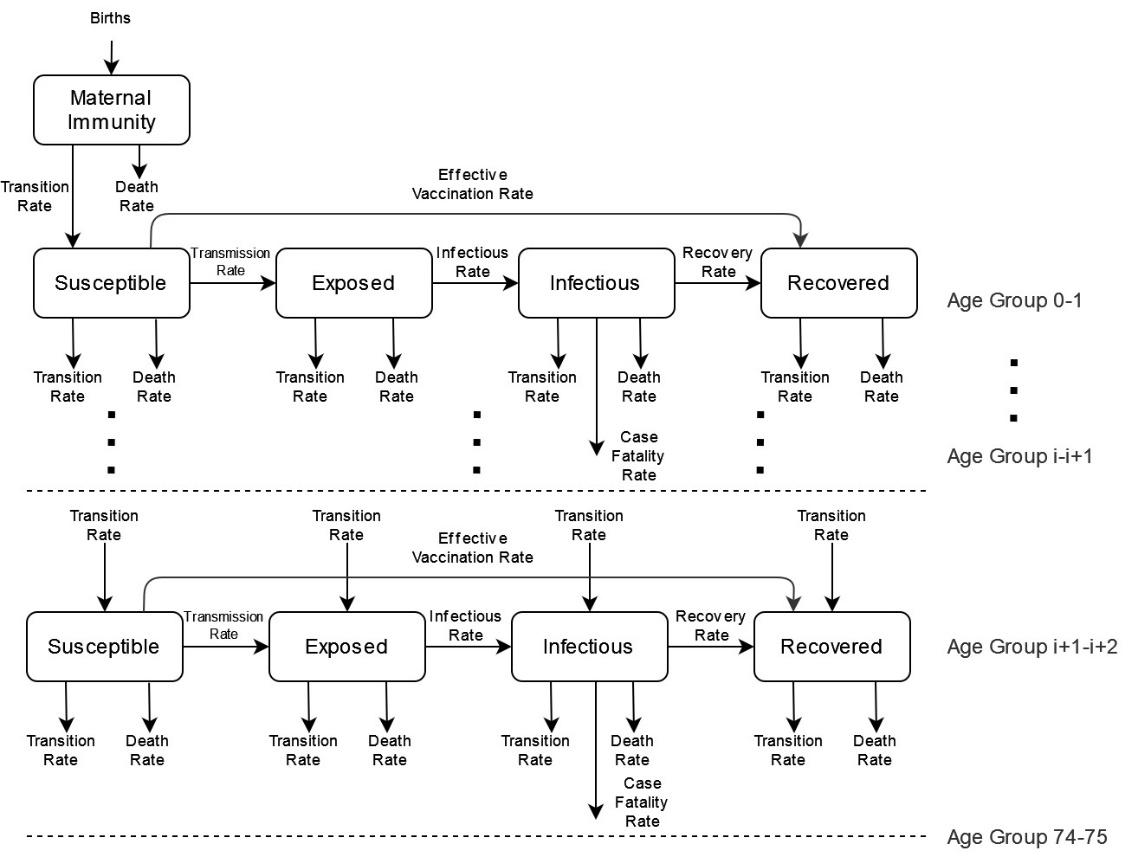


### Model Parameters

**Table A in S1 Appendix.** Disease model parameters.

| **Parameter** | **Description** | **Value** |
| --- | --- | --- |
| Average Force of Infection (𝜆) | The average force of infection for individuals in each age-group (cases per 1,000 people) | Country-dependent, see Table B in S1 Appendix. |
| Leaving exposed rate (𝜎) | Rate at which individuals leave the exposed state and become infectious | 0.1 per day [1] |
| Recovery rate (𝛾) | Rate at which individuals recover from infection | 0.0909 per day [1] |
| Vaccine efficacy (𝜉) | Efficacy of the vaccine | 95% [1] |
| Effective vaccination Coverage (ν) | Immunization coverage at a certain time in percentage. | See Table C in S1 Appendix. |
| CRS probability (𝜌) | The probability that a developing child of an infected mother develops CRS | 65% within the first 16 weeks of pregnancy, 0% thereafter [1] |
| Transition rate (δ) | Transition rate between age groups | Age-dependent. |
| Mortality rate (μ) | Mortality rate for each age group. | Country and age dependent. |
| Time step ($\Delta$) | Discrete time step for Euler’s method. | 1 day. |
| Imported cases (τ) | Number of imported active cases. | 10 cases |
| Births rate (b) | Crude birth rate | Country-dependent. |
| Routine coverage ($c_{k}$) | Routine immunization coverage of dose $k$ | Country-dependent |
| SIA coverage ($c_{SIA}$) | Immunization coverage from SIAs | 80% |

**Table B in S1 Appendix.** Mean Force of Infection (FOI) estimates for Young age (<= 13 years old) and Old age (>13 years old) by country.

| **Country** | **Estimated young mean FOI** | **Estimated old mean FOI** |
| --- | --- | --- |
| AFG | 120.5 (100.5, 140.5) | 23 (0, 57) |
| COD | 130 (87.5, 173.5) | 25 (0, 70.5) |
| ETH | 137 (29, 241) | 99 (0, 295) |
| NGA | 131 (113, 162) | 131 (113, 162) |
| PAK | 144 (122, 161) | 8 (0 ,28) |

**Note:** 95% Confidence intervals presented in parentheses.

**Table C in S1 Appendix.** Routine vaccination coverages 2019 as reported by WHO, 2020.

| **Country** | **MCV1** | **MCV2** |
| --- | --- | --- |
| AFG | 64% | 39% |
| COD | 57% | 30% |
| ETH | 58% | 41% |
| NGA | 54% | 9% |
| PAK | 75% | 71% |

### Equations

In the following section we present the equations that govern the compartmental model. Equations proposed in [1] are presented using their same mathematical notation. We used Euler's method with a time step size of 1 day to solve the system of equations of the SEIR model. For $t mod 365=0$ (beginning of the year), we have the transition between age groups, vaccination of the population and new births. Considering an age group $j$, with $j=0,1, \ldots, 75$, and gender $g$ the equations are defined as:

$$S_{j,g}\left( t \right)= S_{j-1,g}\left( t-1 \right)+ \Delta(-\lambda(t-1) - \mu-\frac{\nu*\xi}{\Delta})S_{j-1,g}\left( t-1 \right)$$

$$E_{j,g}\left( t \right)= E_{j-1,g}\left( t-1 \right)+ \Delta\left( \lambda\left( t-1 \right){*S}_{j-1,g}\left( t-1 \right)-(\sigma+ \mu){*E}_{j-1,g}\left( t-1 \right) \right)$$

$$I_{j,g}\left( t \right)= I_{j-1,g}\left( t-1 \right)+ \Delta\left( \sigma{*E}_{j-1,g}\left( t-1 \right) -(\gamma+ \mu){*I}_{j-1,g}\left( t-1 \right) \right) + \tau$$

$$R_{j,g}\left( t \right)= R_{j-1,g}\left( t-1 \right)+ \Delta\left( \gamma{*I}_{j-1,g}\left( t-1 \right)+\frac{\nu*\xi}{\Delta}*S_{j-1,g}\left( t-1 \right)-\mu{*R}_{j-1,g}\left( t-1 \right) \right)$$

and for $j=0$,

$$S_{0,g}\left( t \right)= E_{0,g}\left( t \right)= I_{0,g}\left( t \right)= R_{0,g}\left( t \right)=0$$

$$M_{0,g}\left( t \right)= b*N$$

For $t mod 180=0$ (first 6 months of a year), we have the transition of 6 months old losing their maternal immunity and being vaccinated. Then, for $j=0$ we define the following equations:

$$S_{0,g}\left( t \right)=\left( 1-\nu\right)* M_{0,g}\left( t-1 \right)$$

$$R_{0,g}\left( t \right)=\nu* M_{0,g}\left( t-1 \right)$$

$$M_{0,g}\left( t \right)= b*N(t-1)$$

For every other $t$, we have:

$$S_{j,g}\left( t \right)= \Delta\left( -\lambda\left( t-1 \right) - \mu\right)S_{j,g}\left( t-1 \right)$$

$$E_{j,g}\left( t \right)= \Delta\left( \lambda\left( t-1 \right){*S}_{j,g}\left( t-1 \right)-(\sigma+ \mu){*E}_{j,g}\left( t-1 \right) \right)$$

$$I_{j,g}\left( t \right)= \Delta\left( \sigma{*E}_{j,g}\left( t-1 \right) -(\gamma+ \mu){*I}_{j,g}\left( t-1 \right) \right)$$

$$R_{j,g}\left( t \right)= \Delta\left( \gamma{*I}_{j,g}\left( t-1 \right)-\mu{*R}_{j,g}\left( t-1 \right) \right)$$

The effective vaccination coverage $\nu$ was determined as follows:

For $j=0$,

$$\nu= max( c_{1}, c_{SIA})$$

for $j=1$, we adjust RCV2 coverage to target only those who got a first dose of RCV1

$$\nu=\left( \frac{c_{1}\left( 1-\xi\right)}{1-c_{1}\xi}c_{2}, c_{SIA} \right)$$

and for $j>1,$

$$\nu= c_{SIA}$$

To calculate the time varying Force of infection, the model uses a contact matrix $C$ which is divided into two age groups: Young (<= 13 years) and Old (> 13 years) is defined as:


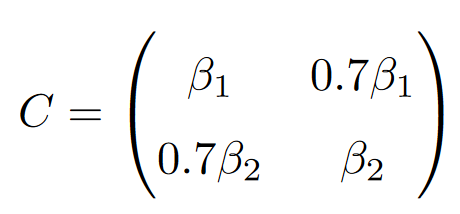


Then, is $\lambda\left( t \right)$ calculated for each group through the following equations:


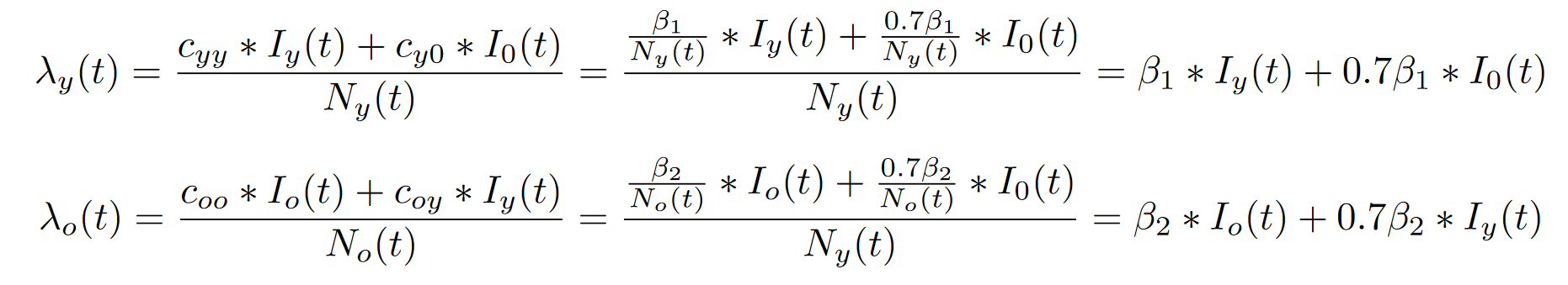


Where,


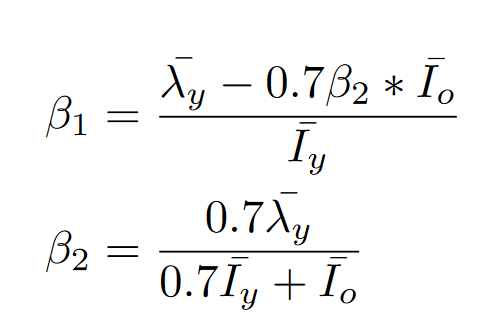


And $\underline{I_{y}}\approx\underline{\lambda}_{y}\underline{S}_{y}D$, $\underline{I_{o}}\approx\underline{\lambda}_{o}\underline{S}_{o}D$ where D is the duration of the disease (11 days). Next, we have that $a_{y}=13$ years and L is the life expectancy of 75 years.


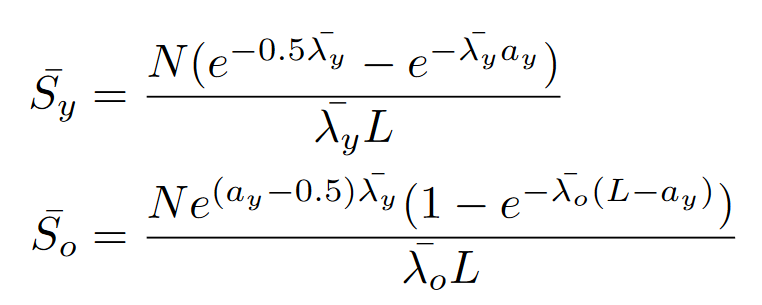


The CRS incidence per 100.000 births is computed using the following equation:


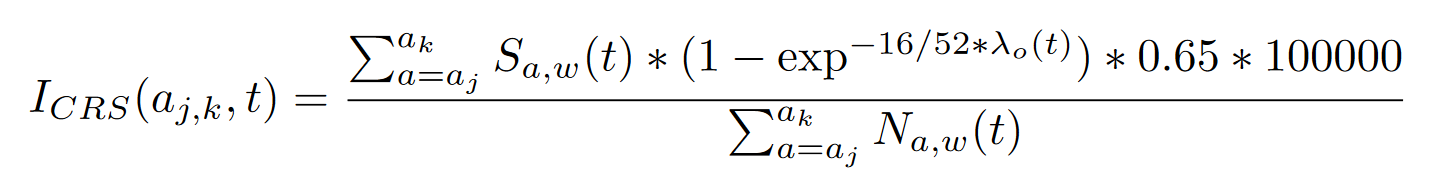


that derives from assuming a 65% probability of a child being born with CRS if the mother was infected during the first 16 weeks of pregnancy.

## Estimates for the Force of Infection

The force of infection parameters were calculated as follows. We used country-level estimates or the single sub-national estimate presented in [1] when available and selected using the first selection criterion. For countries with several sub-national estimates, we calculated the mean and 95% CI using the median values of the parameters available. For countries with no information, we used estimates of countries within the same region, and calculated the mean and 95% CI using the median of those values. The estimates used in these calculations are presented below.

**Table D in S1 Appendix.** Country estimates for Pakistan, Nigeria and Ethiopia

|  | **Pakistan [1]** | **Nigeria [1]** | **Ethiopia [1]** |
| --- | --- | --- | --- |
| FOI young (95% CI) | 144 (122, 161) | 131 (113, 162) | 137 (29,241) |
| FOI old (95% CI) | 8 (0, 28) | 131 (113, 162) | 99 (0, 295) |
| Study, year of data set | [3], 1999-2004 | [5], 2007-08 | [7], 1981 |

**Table E in S1 Appendix.** National force of infection estimates for Afghanistan from regional (Asia, South) estimates.

|  | India [1] | India [1] | India [1] | India [1] | India [1] | Pakistan [1] | Bangladesh [1] | Nepal [1] |
| --- | --- | --- | --- | --- | --- | --- | --- | --- |
| FOI young (95% CI) | 170 (153, 190) | 112 (102, 123) | 129 (99, 158) | 85 (63, 109) | 82 (66. 98) | 144 (122, 161) | 99 (82, 120) | 180 (157, 199) |
| FOI old (95% CI) | 67 (39, 104) | 72 (55, 92) | 27 (0, 85) | 19 (0, 60) | 8 (0, 26) | 8 (0, 28) | 35 (16, 54) | 9 (0, 31) |
| Study, year of data set (region) | [6], 1999-200 (Urban Vellore district) | [6], 1999-2000 (Rural Vellore district) | [8], 1968(Urban Delhi) | [8], 1968 (Rural Delhi) | [9] 1976 (Calcutta) | [3], 1999-2004 | [10], 2004-05 | [11] 2008 |

**Table F in S1 Appendix.** National force of infection estimates for Dem. Republic of Congo from sub-national estimates.

|  | **DRC [2]** | **DRC [2]** | **DRC [2]** | **DRC [2]** |
| --- | --- | --- | --- | --- |
| FOI young (95% CI) | 145 (105, 189) | 103 (66, 138) | 128 (84, 169) | 132 (91, 178) |
| FOI old (95% CI) | 27 (0, 69) | 23 (0, 68) | 20 (0, 73) | 32 (0, 72) |
| Study, year of data set (region) | [4], 2008-09 (City of Kikwit) | [4], 2008-09 (Mikalayi) | [4], 2008-09 (Tshikapa) | [4], 2008-09 (Vanga) |

## Model validation

For model validation we followed the published guidelines for good practices on simulation model validation [12]. Face validity of our results was ensured by external expert opinions. We conducted internal validation by tracking each state in the compartmental model, ensuring that the transitions on each step were done accurately. We also ran synthetic vaccination scenarios and compared results with those expected by literature, e.g. introducing RCV at a high routine coverage level (>80% for both doses) to eradicate Rubella. For cross-validation, because CRS incidence is not available for the countries of interest for 2021, we validated the model by comparing the estimates for CRS incidence obtained in previous studies (see Table G in S1 Appendix). For countries without information, we estimated the CRS incidence estimate using the same procedure as for the force of infection. Our estimates were calculated by running the model with the average force of infection for both young and old populations. The validation results shown represent the mean of the CRS incidence between 2005–2020. The differences between our model and the underlying data are not qualitatively different, residing well within the 95% CIs of each data point.

**Table G in S1 Appendix.** External model validation by comparing CRS incidence results with estimates presented in literature.

|  | **AFG** | **COD** | **ETH** | **NGA** | **PAK** |
| --- | --- | --- | --- | --- | --- |
| Estimate and Source | 65.5  (17, 127)*  Vynnycky et al. (2019) | 66.5  (57, 87)**  Vynnycky et al. (2019)  69  (0, 186)  Alleman et al. (2016) | 95  (0, 253)  (Vynnycky et al. 2016) | 98  (63, 127)  (Vynnycky et al. 2016) | 24  (0, 77)  (Vynnycky et al. 2016) |
| Validation results (absolute difference) | 73.65 [12.4%] | 69.20 [4.1%, 0.3%] | 100.87 [6.18%] | 105.92 [8.1%] | 23.39 [2.5%] |

**Note:** 95% Confidence intervals presented in parentheses. Percent difference from source presented in brackets.

* Estimates from regional data, using the same methodology as in Table E in S1 Appendix.

** Estimates from subnational data, using the same methodology as in Table F in S1 Appendix.

## References

[1] Vynnycky et al., 2016 - Using Seroprevalence and Immunisation Coverage Data to Estimate the Global Burden of Congenital Rubella Syndrome, 1996-2010: A Systematic Review.

[2] Vynnycky et al., 2019 - The impact of Measles-Rubella vaccination on the morbidity and mortality from Congenital Rubella Syndrome in 92 countries.

[3] Ahmed R, Hashmi K, Ullah SE, Khanum T, Rafia A. Study of Prevalence of Immune Status in Adult Females For Rubella Virus Infection. Pakistan Journal of Biological Sciences. 2006;9(5):816.

[4] Alleman MM, Wannemuehler KA, Hao L, Perelygina L, Icenogle JP, Vynnycky E, et al. Estimating the burden of rubella virus infection and congenital rubella syndrome through a rubella immunity assessment among pregnant women in the Democratic Republic of the Congo: Potential impact on vaccination policy. Vaccine. 2016;34(51):6502-11.

[5] Amina MD, Oladapo S, Habib S, Adebola O, Bimbo K, Daniel A. Prevalence of rubella IgG antibodies among pregnant women in Zaria, Nigeria. International Health. 2010;2(2):156-9.

[6] Brown DWJ, Cutts FT, Joseph A. An evaluation of complementary epidemiological methods in a defined population in Southern India for estimating the burden of Congenital Rubella Syndrome. 2004.

[7] Sandow D, Okubagzhi GS, Arnold U, Denkmann N. Seroepidemiological study in rubella in pregnant women in Gondar Region, northern Ethiopia. Ethiopian medical journal. 1982;20(4):173-8.

[8] Seth P, Manjunath N, Balaya S. Rubella infection: the Indian scene. Rev Infect Dis. 1985;7 Suppl 1:S64-7.

[9] Chakravarty MS, Gupta B, Das BC, Mukherjee MK, Mitra AC, Sarkar JK. Seroepidemiological study of rubella in Calcutta. The Indian journal of medical research. 1976;64(1):87-92.

[10] Nessa A, Islam MN, Tabassum S, Munshi SU, Ahmed M, Karim R. Seroprevalence of rubella among urban and rural Bangladeshi women emphasises the need for rubella vaccination of pre-pubertal girls. Indian journal of medical microbiology. 2008;26(1):94-5.

[11] Upreti SR, Thapa K, Pradhan YV, Shakya G, Sapkota YD, Anand A, et al. Developing rubella vaccination policy in Nepal--results from rubella surveillance and seroprevalence and congenital rubella syndrome studies. J Infect Dis. 2011;204 Suppl 1:S433-8.

[12] Eddy DM, Hollingworth W, Caro JJ, Tsevat J, McDonald KM, Wong JB. Model Transparency and Validation: A Report of the ISPOR-SMDM Modeling Good Research Practices Task Force–7. Medical Decision Making. 2012;32(5):733-743. doi:10.1177/0272989X12454579
