# Supplementary material for: Evaluating the potential impact of rubella-containing vaccine introduction on congenital rubella syndrome in Afghanistan, Dem. Republic of Congo, Ethiopia, Nigeria, and Pakistan: A mathematical modeling study
Source: PLOS Glob Public Health. 2024 Jan 16;4(1):e0002656. doi: 10.1371/journal.pgph.0002656 (PMC10791005; doi:10.1371/journal.pgph.0002656)

## Frequency of follow-up SIAs in high-transmission countries

From the previous results we see that NGA and ETH have high variability by the end of the study period. We analyzed the impact in CRS births averted by conducting more frequent SIAs. Fig A in S2 Appendix shows that a significant decrease of CRS births in both countries can be achieved if a follow-up SIA is conducted at least every 3 years (three SIAs at 3, 6 and 9 years after RCV introduction). SIAs play an important role as short-to-medium term actions to avoid the spread of the disease, needing to be conducted at least every 3 years in ETH and NGA over a time span of 10 years to prevent CRS births if the routine immunization remains unchangeable.

**Fig A in S2 Appendix.** CRS births averted under different follow-up SIA frequencies. Annotations show the intervals in years between follow-up SIAs for the first 10 years since introduction.


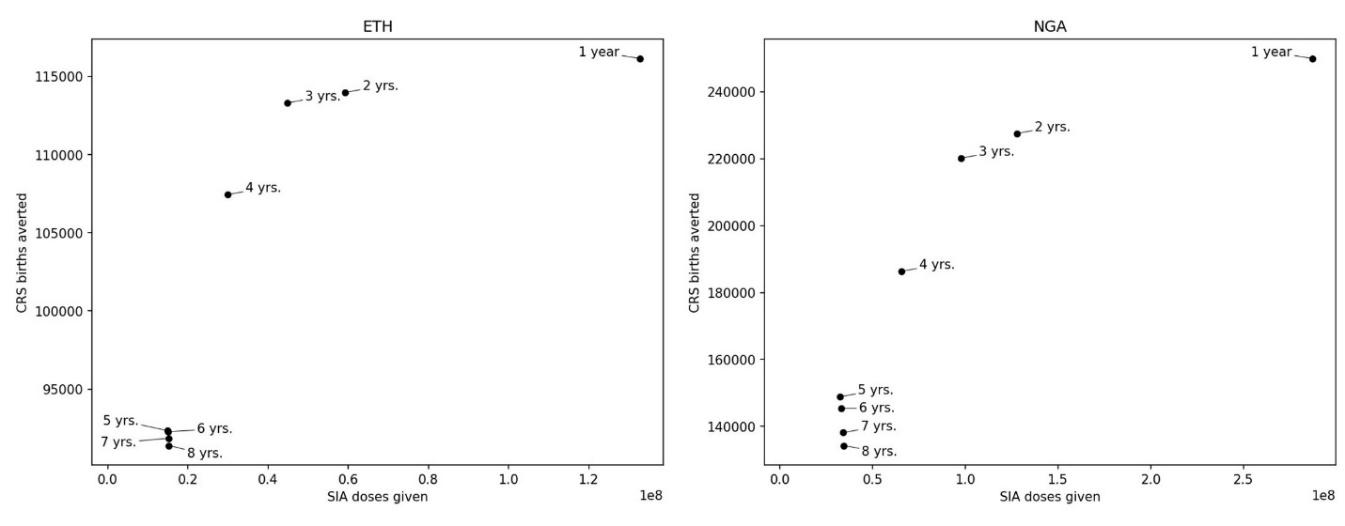

Supplement: S2 Appendix — (DOCX) [file pgph.0002656.s003.docx]
