# Supplementary material for: Evaluating the potential impact of rubella-containing vaccine introduction on congenital rubella syndrome in Afghanistan, Dem. Republic of Congo, Ethiopia, Nigeria, and Pakistan: A mathematical modeling study
Source: PLOS Glob Public Health. 2024 Jan 16;4(1):e0002656. doi: 10.1371/journal.pgph.0002656 (PMC10791005; doi:10.1371/journal.pgph.0002656)

## Projected outcomes and rubella transmission dynamics for each scenario

**Table A in S3 Appendix.** Scenario S0 Projected CRS births and DALYs.

|  | | **AFG** | | **COD** | | **ETH** | | **NGA** | | **PAK** | |
| --- | --- | --- | --- | --- | --- | --- | --- | --- | --- | --- | --- |
| Scenario | Projection | CRS births | DALYs | CRS births | DALYs | CRS births | DALYs | CRS births | DALYs | CRS births | DALYs |
| **S0** | **L** | 21,951 | 636,583 | 83,290 | 2,415,406 | 98,142 | 2,846,124 | 262,977 | 7,889,319 | 34,545 | 1,036,337 |
|  | **M** | 26,659 | 773,122 | 95,109 | 2,758,154 | 117,876 | 3,418,399 | 299,903 | 8,997,099 | 42,265 | 1,267,942 |
|  | **H** | 31,507 | 913,702 | 107,276 | 3,110,996 | 138,241 | 4,008,976 | 337,828 | 10,134,852 | 50,264 | 1,507,916 |

**Fig A.** Transmission of rubella and vaccination coverage in Afghanistan.


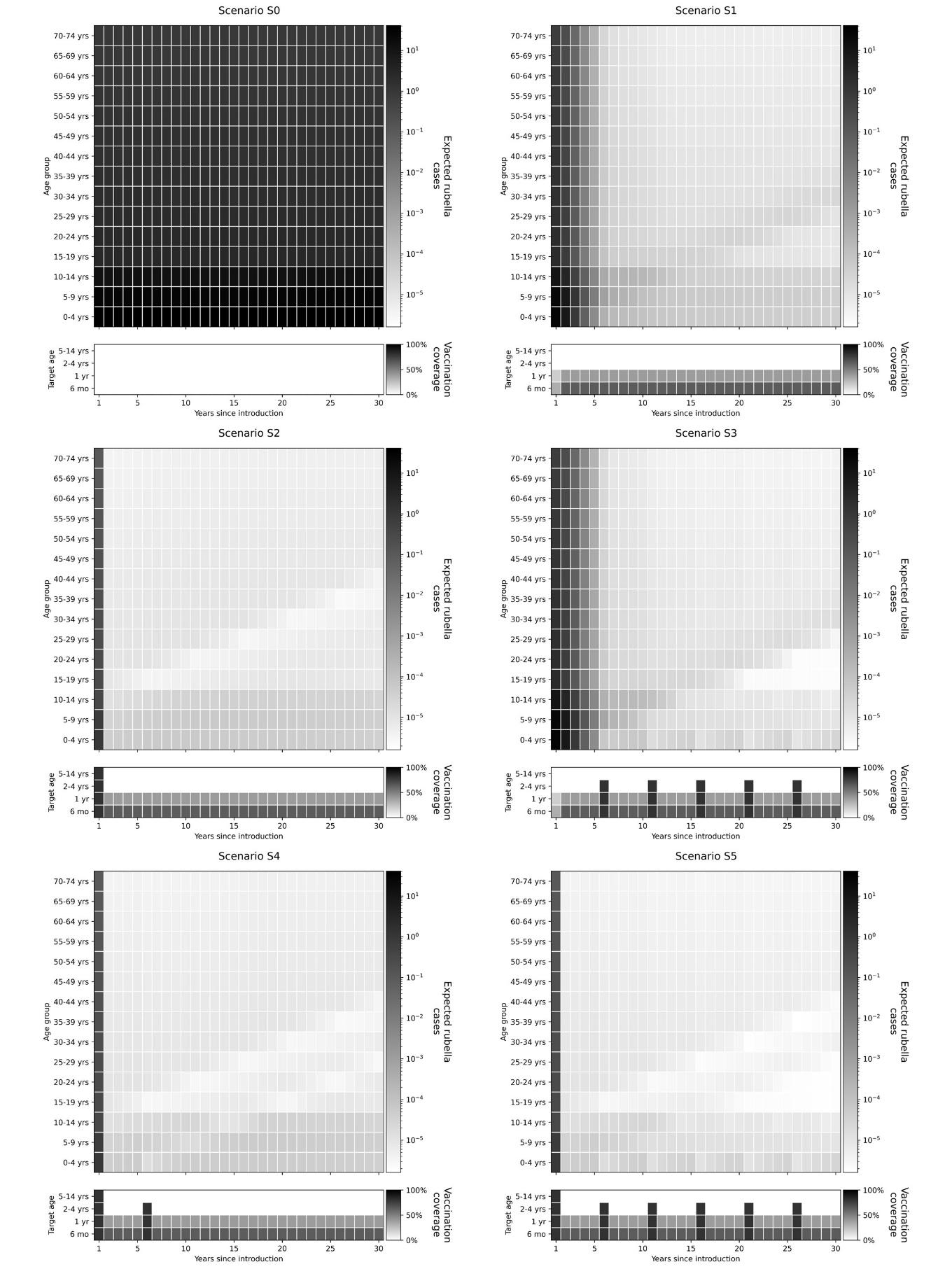


**Fig B.** Transmission of rubella and vaccination coverage in Dem. Republic of Congo.


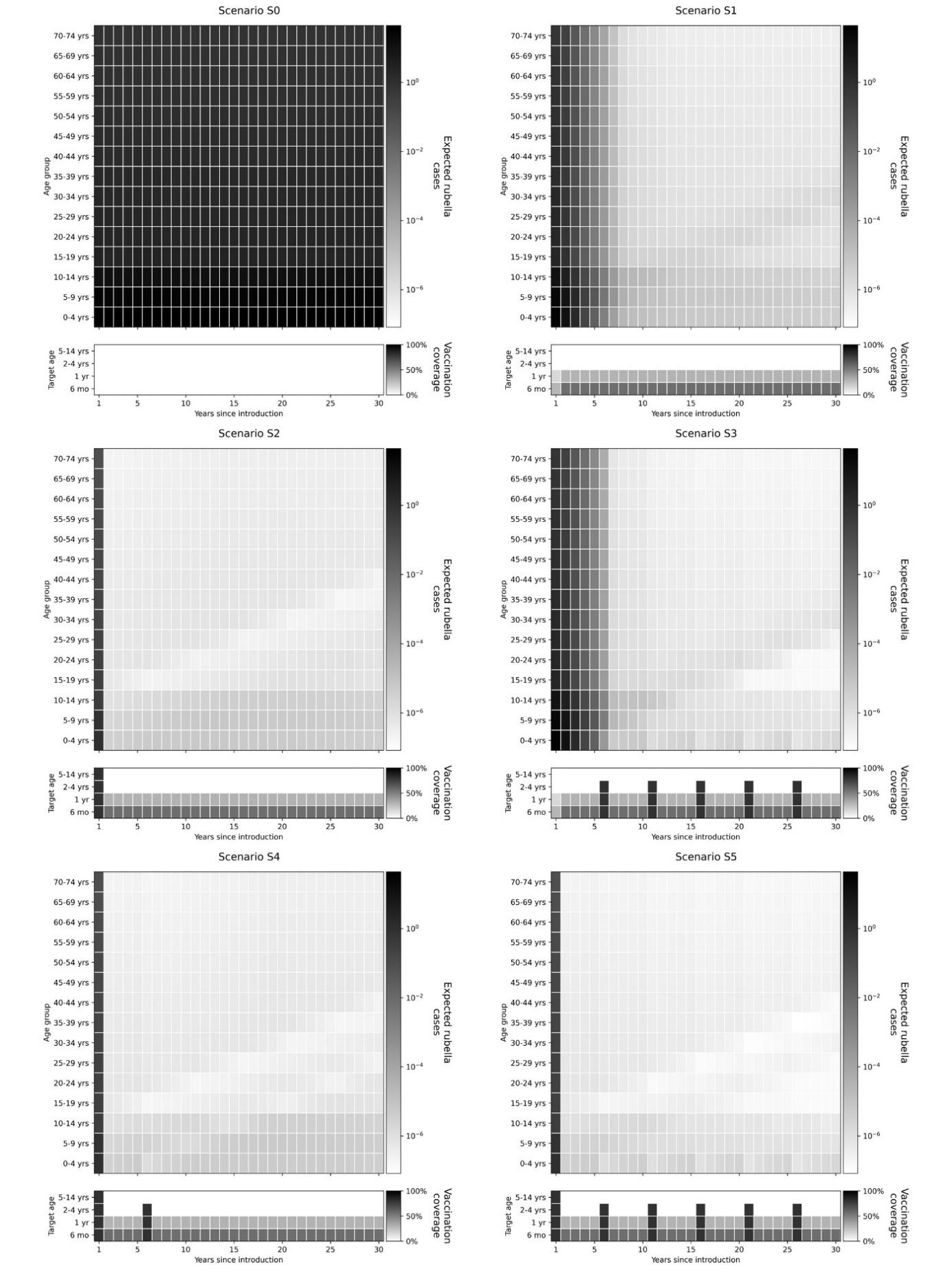


**Fig C.** Transmission of rubella and vaccination coverage in Ethiopia.


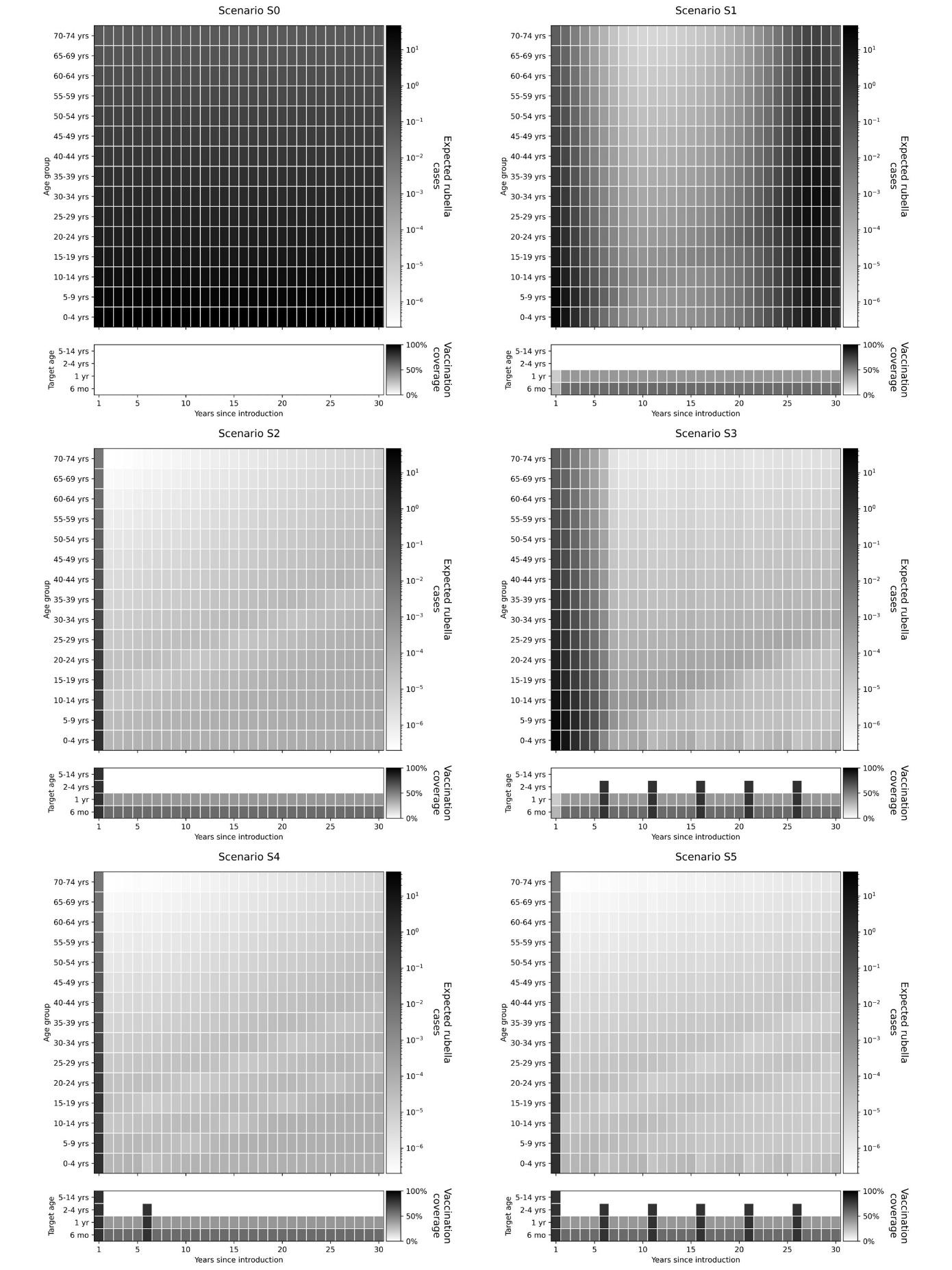


**Fig D.** Transmission of rubella and vaccination coverage in Nigeria.


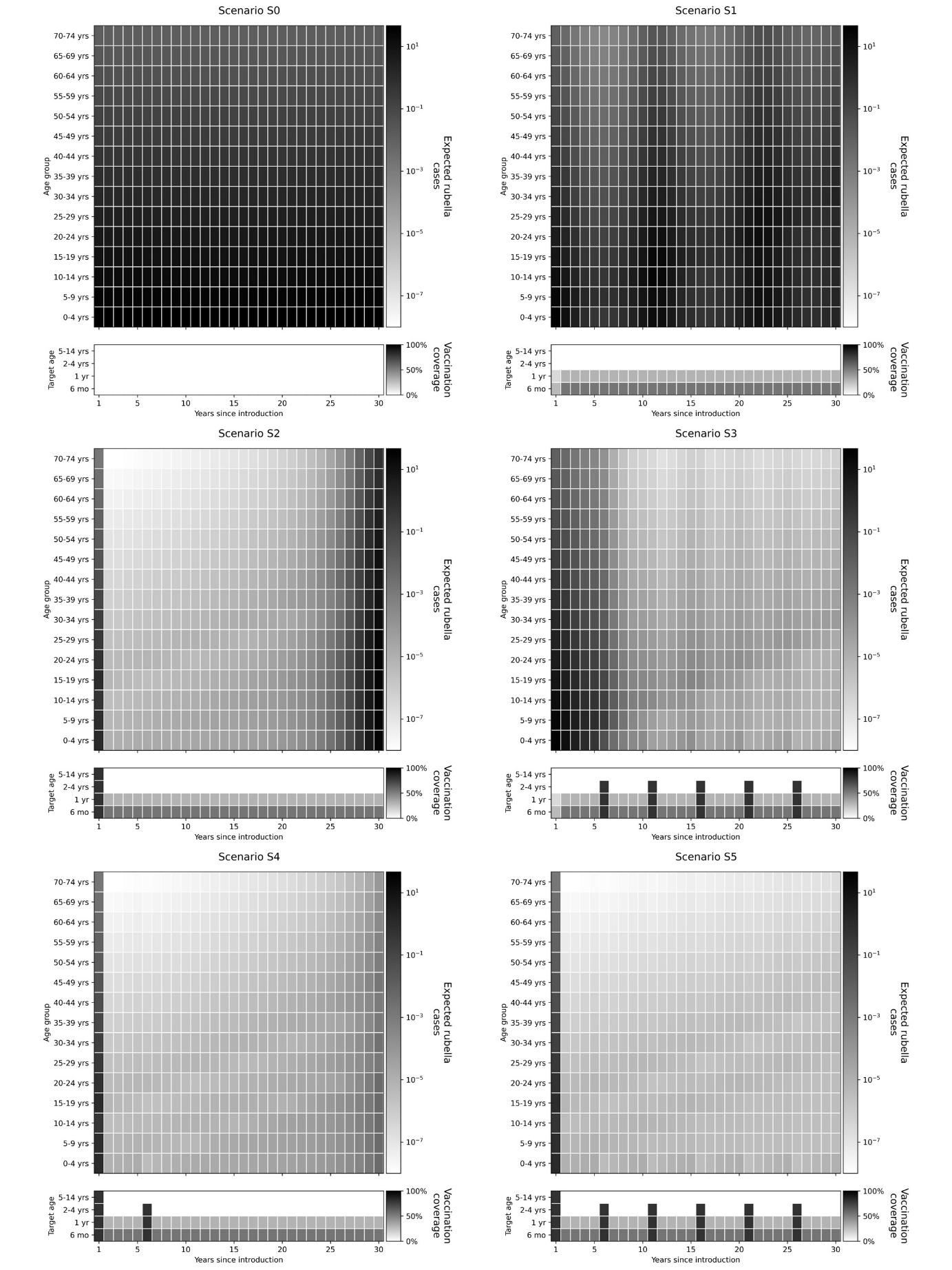


**Fig E.** Transmission of rubella and vaccination coverage in Pakistan.


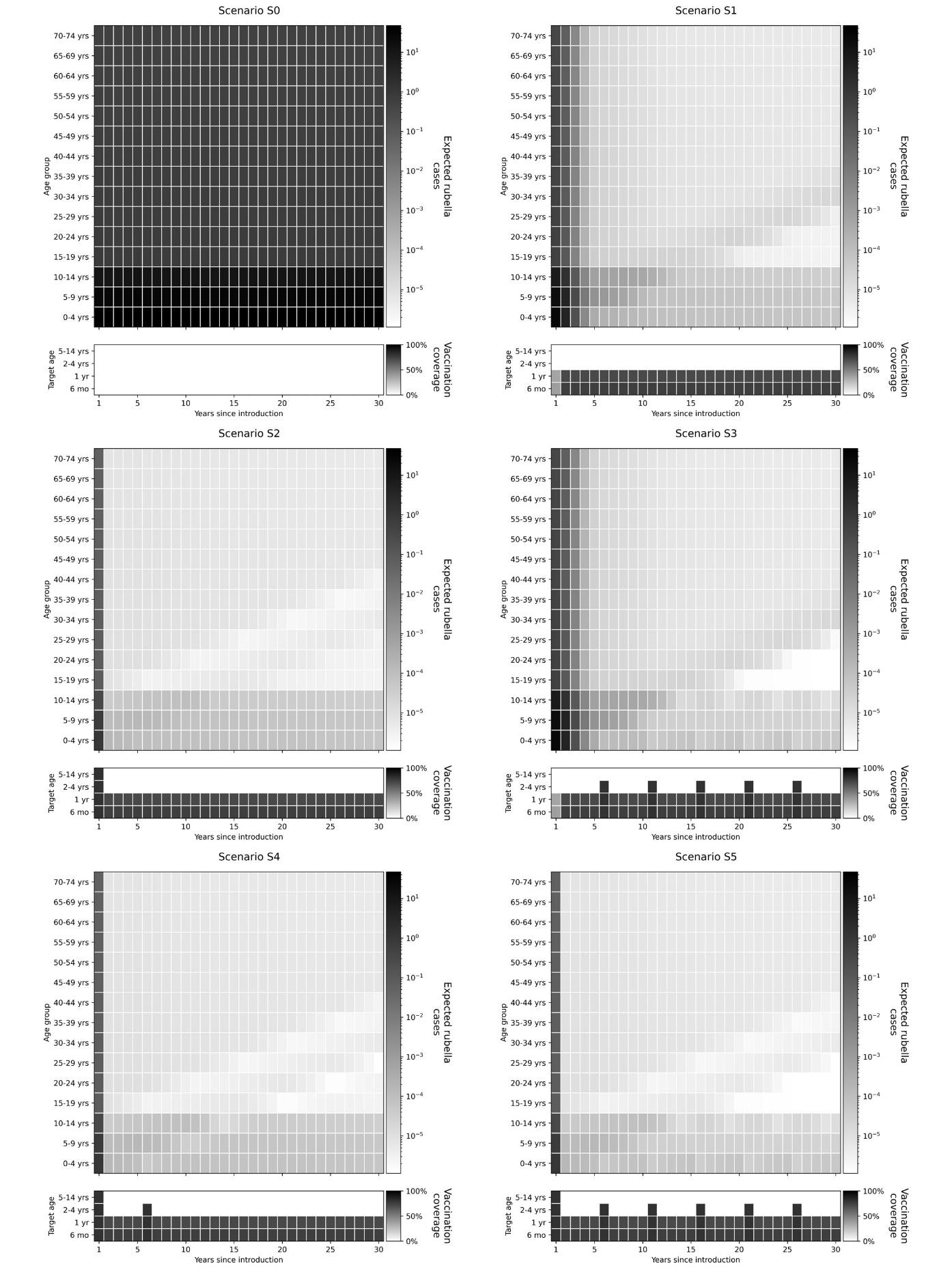

Supplement: S3 Appendix — (DOCX) [file pgph.0002656.s004.docx]
