# Supplementary material for: Evaluating the potential impact of rubella-containing vaccine introduction on congenital rubella syndrome in Afghanistan, Dem. Republic of Congo, Ethiopia, Nigeria, and Pakistan: A mathematical modeling study
Source: PLOS Glob Public Health. 2024 Jan 16;4(1):e0002656. doi: 10.1371/journal.pgph.0002656 (PMC10791005; doi:10.1371/journal.pgph.0002656)
Supplement: Attachment — Submitted filename: Review of PGPH-D-23-01509_reviewer.pdf [file pgph.0002656.s005.pdf]

The investigators report a mathematical modeling study of the estimated potential impact of introduction and use of RCV on the occurrence of CRS over 30 years in five countries currently not vaccinating against rubella. They used a catalytic compartment differential equation-based model with states, M, S, E, I, and R. They assumed maternal antibody, which would prevent immunization by killing the RCV virus, is gone by 6 months of infant age. They assumed a 2-dose RCV routine schedule following introduction, with the first dose after 6 months, timed to coincide with MCV (as a combination vaccine). Country-specific RCV coverage levels were capped at MCV coverage levels reported to WHO/UNICEF (or 30% if MCV2 has not been introduced), with ramp-up from 0% to 100% of MCV coverage after two years. Total effective vaccination was assumed to be the product of the vaccine efficacy (0.95, the seroconversion rate) and the maximum between routine coverage and an assumed SIA coverage of 80%. They modeled five vaccination scenarios that were combinations of routine vaccination and SIAs (a wide-age-ranging SIA among 0-14-year-olds and follow-up SIAs among 0-4-year-olds) and one scenario of no RCV introduction, under assumptions of three different birth rates. Model outcomes were CRS cases and DALYs. They modeled various coverage levels to determine a coverage level in which CRS decreases. They conducted sensitivity analyses of the FoI from their catalytic model. They found that with the exception of one country under the scenario of RI-only (in which CRS increases due to RCV introduction), introduction of RCV decreases CRS substantially, varying by scenario. Three countries with high MCV coverage may only need SIAs around RCV introduction time; two other countries need SIAs for an indeterminate period. Sensitivity analysis showed that RI and SIA coverage needs to be improved beyond current levels to prevent future CRS outbreaks. They conclude that (proper) RCV introduction reduces CRS, that a second RCV dose is necessary in countries with 1-dose coverage is less than 80%, and that RI coverage needs to be increased.

The topic is of obvious importance as all WHO regions have a goal to eliminate rubella (and therefore prevent CRS). Mathematical modeling is the most common way of predicting vaccine impact on rubella and CRS. Their use of a catalytic compartment model is appropriate. Their scenarios make programmatic sense. Use of current MCV coverage data is appropriate although conservative. The manuscript is clearly written. Between the manuscript and the supplementary file, their model is described quite well. Their conclusions are based on model outputs.

I have a few suggestions to improve this quite good manuscript.

The title, "Evaluating the impact of rubella-containing vaccine introduction in congenital rubella syndrome for Afghanistan, Dem. Republic of Congo, Ethiopia, Nigeria, and Pakistan," makes it appear that this is a study of the actual impact of RCV introduction. However, it is a study of the potential impact of RCV introduction based on a mathematical model. STOBE guidelines suggest putting the study design in the title. I think that their title should be "Evaluating the potential impact of … : a mathematical modeling study."

Throughout the manuscript, when appropriate, the authors should make sure that it is clear that impact refers to potential impact. For example, the first sentence in the Abstract, "we assessed the impact of introducing rubella-containing vaccine" should be changed to indicate to the reader that it is potential impact.

The scenario in which RCV is never introduced is used to estimate the reduction of CRS births in the vaccinating scenarios. It would help the reader to put the CRS reduction values in context if the number of CRS births in the non-vaccinating scenario was included in the results – in the narrative and especially

in Table 2 of the manuscript. Reporting the base case number of CRS cases would help the reader evaluate the face validity of the model. Additionally, when appropriate, a percentage reduction of CRS (in addition to a numeric reduction) would be a meaningful result for the reader to interpret.

On Line 133, “endemic equilibrium” is mentioned as a starting condition for the study. However, endemic equilibrium is not defined. Rubella occurs in periodic epidemics, which are likely to occur in different times in the five countries. The authors should define what they mean by endemic equilibrium.

The authors used two doses in RI for all scenarios other than the non-vaccination scenario. Since the take rate of a single dose of RCV is >95% and believed with some evidence to be lifelong, a second dose only immunizes the few who didn’t seroconvert from one dose. It would make more policy sense to put the effort into giving a first dose to more children than revaccinating the immune. Such an effort is consistent with the WHO Immunization Agenda 2030 strategy of having no zero-dose children. The problem addressed in the countries requiring a second dose is low coverage (lines 225-233). The authors recommend continued SIAs, but a far better strategy would be to strengthen RI to reach more children, reducing zero-dose children and achieving immunization benefits from more than just rubella vaccine. The SIAs, presumably, are to reach the unvaccinated children, but as the authors point out (lines 128-132), most children vaccinated in SIAs are already immune through RI. Since WHO, through IA2030, is promoting RI to reduce zero-dose children, it would be good to have at least one scenario in which RI improves.

The authors conclusion that two RCV doses is required for certain countries goes against the WHO recommendation of needing only a single dose. Therefore, further explanation of why two doses are required would help the reader.

The authors determination that effective vaccination was the product of the one-dose take rate and the maximum between RI coverage and SIA coverage seems a bit artificial (lines 129-131). There is an assumption in their approximation of which children are vaccinated in follow-up SIAs. They authors should indicate the thinking behind their method to estimate effective vaccination.
