# Supplementary material for: Evaluating the potential impact of rubella-containing vaccine introduction on congenital rubella syndrome in Afghanistan, Dem. Republic of Congo, Ethiopia, Nigeria, and Pakistan: A mathematical modeling study
Source: PLOS Glob Public Health. 2024 Jan 16;4(1):e0002656. doi: 10.1371/journal.pgph.0002656 (PMC10791005; doi:10.1371/journal.pgph.0002656)
Supplement: Attachment — Submitted filename: Response to reviewers.pdf [file pgph.0002656.s006.pdf]

Manuscript PGPH-D-23-01509

Response to reviewers

PLOS Global Public Health

Dear Editorial board,

Thank you for allowing us the opportunity to revise our paper. The review team has provided insightful remarks and constructive criticisms, which have been incorporated into the revised manuscript. In what follows, we provide a point-by-point response to each reviewer's comments. The reviewers' comments appear in italics followed by our responses in normal text. Numbering has been added to reviewer comments for ease of reference. We have also attached a separate version of the manuscript (Tracked Changes Version) where the changes we made appear in blue font, so that the revisions can be easily identified. Please note, line numbers referenced in the response refer to line numbers in the Tracked Changes version. Because of the review team's suggestions, the paper is much improved in terms of practical applicability and contributions to the academic literature, and we thank the reviewers and editors for taking the time to read our paper and provide helpful feedback.

The main changes made to the manuscript are:

- Thanks to the feedback provided by the reviewers, we revised our assumption for how we treated RCV2 in the model. We changed our assumptions to consider that a second dose was applied only to those children that initially were given an RCV1 dose. The modifications to the mathematical equations are highlighted in the supplementary information file. We updated our numerical results based on this modification, and the majority of our conclusions remain inline with those presented in the original manuscript.
- We incorporate reviewers' feedback regarding the promotion of policies considering WHO's recommendations. We highlight the need to increase routine coverage while also conducting the recommended supplemental immunization activities. Additionally, we discuss how limitations of the model can result in discrepancies from those recommendations.
- We included additional limitations of the study pointed out by the reviewers, and discussed how they affect the potential impacts estimated for the studied countries.
- In addition to changes related to comments from reviewers, we have used colors (instead of grayscale) in all figures in the manuscript to enhance interpretability of results.
- We cited an additional study to support the limitation pointed out by Reviewer 3 in comment 7 related to the impact of the quality of reported vaccination coverage on our outcomes.

Reviewer #1: *This is an interesting paper addresses rubella vaccination and its impact on CRS. Here are a few comments that could improve on the manuscript:*

1. *I think the title can be improved. It currently reads "Evaluating the impact of rubella-containing vaccine introduction in congenital rubella syndrome for Afghanistan, Dem. Republic of Congo, Ethiopia, Nigeria, and Pakistan" but should be modified to read "Evaluating the impact of rubella-containing vaccine introduction on congenital rubella syndrome in Afghanistan, Dem. Republic of Congo, Ethiopia, Nigeria, and Pakistan"*

We thank the reviewer for pointing this out. Also considering comment 1 from Reviewer 2 we have changed our title to: **Evaluating the impact of rubella-containing vaccine introduction on congenital rubella syndrome in Afghanistan, Dem. Republic of Congo, Ethiopia, Nigeria, and Pakistan: a mathematical modeling study.**

2. *There are minor language errors throughout the manuscript which a copy editor can address.*

We thank the reviewer for bringing this to our attention. We have revised the manuscript and made amendments accordingly. Small changes (e.g., typos) are not highlighted in the Track Changes document.

3. *In the methods section, you assumed that the first year of introduction the RCV coverage would correspond to 50% of the MCV immunization coverage and kept constant at 100% for the following years of the planning. I find this surprising because usually there is a lot of risk communication and community engagement prior to introduction of new vaccines which is likely to increase the coverage levels for the measles and rubella combination vaccine. Please provide an explanation for the assumption that RCV coverage will be lower than the figures for MCV in the first year of introduction.*

We acknowledge the reviewer's comment regarding the impact of additional efforts at the time of introduction. However, we made this conservative assumption based on several factors. First, we looked at the reported RCV introduction coverage of countries with similar MCV coverage as the studied countries, and we found that in some cases the same coverage was not achieved. Second, we know that in practice introduction of RCV could happen any time during a year, and therefore, some proportion of the target cohort might not have been immunized within a calendar year. Third, incorporating a new vaccine is paired with logistic challenges. The availability of the vaccine might not be guaranteed in all sub-national regions at the same time, and we incorporate this uncertainty through an initial decrease in the initial routine coverage. All in all, the assumption was made to be conservative with the potential impact of a successful RCV introduction.

4. *In lines 241-243 of the results, you write " Countries with high transmission levels (NGA and ETH) require a 95% introduction rate to ensure sustained reduction in CRS*

incidence, with routine coverage below 80% resulting in late outbreaks." It is surprising that a coverage figure of 95% is required to obtain a sustained reduction in CRS incidence. Rubella is not as transmissible as measles so it would be helpful to further justify this finding.

We acknowledge that rubella is not as transmissible as measles. In our model, the time-varying force of infection depends on the number of infected individuals at a certain moment in time and on the underlying average force of infection of each country (see Supplemental information A). One limitation of our model is that it assumes that the underlying average force of infection (FOI) for each country remains constant. Therefore, long-term changes to the underlying average FOI due to a higher proportion of vaccinated people is not being captured by the model. Hence, the high transmission profiles (NGA and ETH) remain high for the entire period of study, needing a higher routine coverage to prevent CRS cases. We included this limitation in the Discussion section (see lines 292-301, 339-340).

Furthermore, to test the impact of this assumption we conducted a sensitivity analysis around the underlying average FOI. The sensitivity analysis suggests that for a wide range of average force of infection values we would still expect high CRS incidence by the end of the study period (see lines 263-274).

5. *In the results section, I find it difficult to distinguish the five scenarios in figure 1. In addition, the figures look blurred and the writings are not clearly visible.*

We have addressed this by adding color to our figures instead of a grayscale. We have also made sure that figures are compliant with PLOS quality requirements to avoid the highlighted issues.

6. *In lines 284-285, you write "A second dose of RCV is required, especially in those countries where current first-dose coverage is below the recommended target of 80%". I think this needs a bit more explanation given that the second dose of RCV is likely to be administered to the same group of children who received the first dose and not those who missed the first dose.*

Our previous assumption overestimated the potential impact of a second dose of RCV in averting CRS births. In the revised results and discussion sections, we have updated the numerical results to account for an RCV2 that is administered to those children who already received a first dose (see lines 233-245, 302-311). Results show that there exists a marginal benefit in incorporating RCV2 to the immunization schedule, especially in those countries with relatively low RCV1 coverage. Although the number of averted cases is lower than those we previously estimated, results highlight that there is a potential benefit of also including a combination vaccine for the second dose as a complementary action while the country ramps-up its RCV1 coverage to the WHO recommended level.

7. *It would be helpful to discuss how changes in population immunity following vaccine introduction affect the projections of CRS incidence over time. Do the estimates of force of infection remain constant over the entire simulation period irrespective of the vaccination coverage achieved?*

We thank the reviewer for this comment. As mentioned in comment #4 above, a limitation of our model is that it assumes a constant underlying average force of infection for each country, and therefore, it does not capture the reduction in it due to a higher proportion of vaccinated people. Nonetheless, cases are being driven by the time-varying force of infection that depends on the average force of infection and the number of infected individuals (see Supplemental information A). We have added this limitation to the Discussion section (see lines 292-301, 339-340). Moreover, we quantified part of the uncertainty related to the average force of infection estimates in the sensitivity analysis, which shows the potential CRS incidence for different infectivity levels (see lines 263-274).

8. *I think an additional limitation is the fact that sub-national variation in rubella epidemiology are not accounted for.*

We agree with the reviewer that this is an important limitation to discuss, and we have added this to our Discussion section (see lines 342-343).

Reviewer #2: *The investigators report a mathematical modeling study of the estimated potential impact of introduction and use of RCV on the occurrence of CRS over 30 years in five countries currently not vaccinating against rubella. They used a catalytic compartment differential equation-based model with states, M, S, E, I, and R. They assumed maternal antibody, which would prevent immunization by killing the RCV virus, is gone by 6 months of infant age. They assumed a 2-dose RCV routine schedule following introduction, with the first dose after 6 months, timed to coincide with MCV (as a combination vaccine). Country-specific RCV coverage levels were capped at MCV coverage levels reported to WHO/UNICEF (or 30% if MCV2 has not been introduced), with ramp-up from 0% to 100% of MCV coverage after two years. Total effective vaccination was assumed to be the product of the vaccine efficacy (0.95, the seroconversion rate) and the maximum between routine coverage and an assumed SIA coverage of 80%. They modeled five vaccination scenarios that were combinations of routine vaccination and SIAs (a wide-age-ranging SIA among 0-14-year-olds and follow-up SIAs among 0-4-year-olds) and one scenario of no RCV introduction, under assumptions of three different birth rates. Model outcomes were CRS cases and DALYs. They modeled various coverage levels to determine a coverage level in which CRS decreases. They conducted sensitivity analyses of the FoI from their catalytic model. They found that with the exception of one country under the scenario of RI-only (in which CRS increases due to RCV introduction), introduction of RCV decreases CRS substantially, varying by scenario. Three countries with high MCV coverage may*

only need SIAs around RCV introduction time; two other countries need SIAs for an indeterminate period. Sensitivity analysis showed that RI and SIA coverage needs to be improved beyond current levels to prevent future CRS outbreaks. They conclude that (proper) RCV introduction reduces CRS, that a second RCV dose is necessary in countries with 1-dose coverage is less than 80%, and that RI coverage needs to be increased.

The topic is of obvious importance as all WHO regions have a goal to eliminate rubella (and therefore prevent CRS). Mathematical modeling is the most common way of predicting vaccine impact on rubella and CRS. Their use of a catalytic compartment model is appropriate. Their scenarios make programmatic sense. Use of current MCV coverage data is appropriate although conservative. The manuscript is clearly written. Between the manuscript and the supplementary file, their model is described quite well. Their conclusions are based on model outputs.

I have a few suggestions to improve this quite good manuscript.

1. *The title, "Evaluating the impact of rubella-containing vaccine introduction in congenital rubella syndrome for Afghanistan, Dem. Republic of Congo, Ethiopia, Nigeria, and Pakistan," makes it appear that this is a study of the actual impact of RCV introduction. However, it is a study of the potential impact of RCV introduction based on a mathematical model. STOBE guidelines suggest putting the study design in the title. I think that their title should be "Evaluating the potential impact of ... : a mathematical modeling study."*

We thank the reviewer for pointing this out. Also considering comment 1 from Reviewer 1 we have changed our title to: **Evaluating the impact of rubella-containing vaccine introduction on congenital rubella syndrome in Afghanistan, Dem. Republic of Congo, Ethiopia, Nigeria, and Pakistan: a mathematical modeling study.**

2. *Throughout the manuscript, when appropriate, the authors should make sure that it is clear that impact refers to potential impact. For example, the first sentence in the Abstract, "we assessed the impact of introducing rubella-containing vaccine" should be changed to indicate to the reader that it is potential impact.*

We agree with the reviewer that this is the correct way of referring to our results and we have changed the manuscript accordingly.

3. *The scenario in which RCV is never introduced is used to estimate the reduction of CRS births in the vaccinating scenarios. It would help the reader to put the CRS reduction values in context if the number of CRS births in the non-vaccinating scenario was included in the results – in the narrative and especially in Table 2 of the manuscript. Reporting the base case number of CRS cases would help the reader evaluate the face*

validity of the model. Additionally, when appropriate, a percentage reduction of CRS (in addition to a numeric reduction) would be a meaningful result for the reader to interpret.

We thank the reviewer for this useful feedback. We included the percentage of CRS births averted relative to the no vaccination scenario (S0) in Table 2. We decided to include the estimated CRS births for the latter in the Supporting information file (see Table D.1), as it would increase the length of an already long table. Additionally, we included the percentage reduction in CRS births in several sections to facilitate the interpretation of results.

4. *On Line 133, "endemic equilibrium" is mentioned as a starting condition for the study. However, endemic equilibrium is not defined. Rubella occurs in periodic epidemics, which are likely to occur in different times in the five countries. The authors should define what they mean by endemic equilibrium.*

We agree that this was not clear in the manuscript. We refer to endemic equilibrium as the asymptotically stable state achieved by the compartmental model when the disease is not eradicated from a population given a specific average force of infection[1]. We have added this definition in Section 2.2. (see lines 135-137).

5. *The authors used two doses in RI for all scenarios other than the non-vaccination scenario. Since the take rate of a single dose of RCV is >95% and believed with some evidence to be lifelong, a second dose only immunizes the few who didn't seroconvert from one dose. It would make more policy sense to put the effort into giving a first dose to more children than revaccinating the immune. Such an effort is consistent with the WHO Immunization Agenda 2030 strategy of having no zero-dose children. The problem addressed in the countries requiring a second dose is low coverage (lines 225-233). The authors recommend continued SIAs, but a far better strategy would be to strengthen RI to reach more children, reducing zero-dose children and achieving immunization benefits from more than just rubella vaccine. The SIAs, presumably, are to reach the unvaccinated children, but as the authors point out (lines 128-132), most children vaccinated in SIAs are already immune through RI. Since WHO, through IA2030, is promoting RI to reduce zero-dose children, it would be good to have at least one scenario in which RI improves.*

We acknowledge that our previous assumption overestimated the potential impact of a second dose of RCV in averting CRS births. In the revised results and discussion sections, we have updated the numerical results to account for an RCV2 that is administered to those children who already received a first dose (see lines 233-245, 302-311). Our updated results show that even though there is a marginal benefit in incorporating a second dose of RCV, the greater impact is seen when RCV1 coverage is increased to WHO recommended level, as you have intuited. We

---

[1] [1]Brauer F, Castillo-Chavez C, Feng Z. Endemic Disease Models. Mathematical Models in Epidemiology. 2019 Jun 25;69:63–116. doi: 10.1007/978-1-4939-9828-9_3. PMCID: PMC7316091.

point out that RCV2 can be a complementary action to be taken (especially with the introduction of combination vaccines) while a country increases its RCV1 coverage.

We also appreciate the feedback regarding including a scenario where routine immunization improves. Currently, we lack information on how much the increase in routine immunization should be for each country. However, the revised results shown in Section 3.3 show the impact of an increment in RCV1 vaccination if we consider a catch-up SIA and a single SIA conducted after 5 years of introduction (see lines 249-259, 292-301). We show the decrease in CRS incidence as RCV1 coverage level is increased to different levels, and we compared these results with the recommended WHO guidelines.

6. *The authors conclusion that two RCV doses is required for certain countries goes against the WHO recommendation of needing only a single dose. Therefore, further explanation of why two doses are required would help the reader.*

We thank the reviewer for the feedback. While a second dose might be useful to prevent CRS births when there is low coverage of RCV1, we acknowledge that to be better aligned to the WHO recommendation the takeaway should be that RCV2 can be a complementary action to be made while a country increases its RCV1 coverage. To highlight this, in the revision of the manuscript we reframed the experiment to assess the potential impact of not including RCV2 in the immunization schedule.

The word "required" was also too strong, it is no longer included in our recommendations. Instead, we point out that RCV2 can be a complementary action to prevent CRS cases while a country increases its RCV1 coverage.

7. *The authors determination that effective vaccination was the product of the one-dose take rate and the maximum between RI coverage and SIA coverage seems a bit artificial (lines 129-131). There is an assumption in their approximation of which children are vaccinated in follow-up SIAs. They authors should indicate the thinking behind their method to estimate effective vaccination.*

Thanks for pointing out that this statement needs clarification. The rationale behind this assumption is that SIAs are likely to reach children who are up-to-date with routine vaccination. Therefore, for those age groups that are part of the routine vaccination schedule, we assumed that if SIA coverage is less than the routine coverage, then those supplementary doses are given to children who already got a routine RCV dose. On the other hand, if SIA coverage is higher than that of routine, we assumed that the age group is vaccinated at the SIAs immunization rate. For simplicity, we assumed that only one dose is given to each child in those specific age groups at a time. Hence, by taking the maximum between the routine and SIA coverage, we are considering that either a child gets a single routine dose or a single SIA dose. We rephrase this in Section 2.2 for clarification (see lines 130-133).

Reviewer #3: *This modeling study examined the impact of introducing a rubella-containing vaccine (RCV) on congenital rubella syndrome (CRS) incidence in Afghanistan (AFG), Democratic Republic of Congo (COD), Ethiopia (ETH), Nigeria (NGA), and Pakistan (PAK). It is a relevant study that could potentially guide the vaccination strategies that could be deployed in different endemic countries yet to introduce an RCV-containing vaccine given different underlying conditions and assumptions. It is well-written but still requires minor editing. The limitations were documented but not exhaustive.*

Specific comments:

1. *Kindly revisit this sentence: "As of 2021, all these countries have MCV1 (1st dose of measles-containing vaccine) as part of their routine vaccination schedule, and only the Democratic Republic of Congo is yet to report introduction coverage of MCV2". Seems there is no congruency.*

We thank the reviewer for pointing this out. We have clarified this phrase in the Introduction section (see lines 59-62) as follows:

"Moreover, all these countries have included MCV1 and MCV2 (the first and second doses of measles-containing vaccine) in their routine vaccination schedules, except for the Democratic Republic of Congo, which has not included MCV2."

2. *You may want to provide additional potential gains from the study (not limited to this): "Results can be used to better inform resource allocation decisions toward 82 the elimination of rubella"*

We added the following potential gain of the study to the Introduction Section, which relates to the purpose of the study (see lines 82-86):

"Results serve as guidance for policymakers aiming to incorporate RCV into their immunization schedules, considering the current routine coverage levels in each country, and to better inform decisions regarding resource allocation towards the eradication of CRS."

3. *We also assumed that SIA campaigns are conducted at the beginning of each year. We also adapted the model to introduce 10 cases per year to incorporate the possibility of imported cases. What guided these assumptions- may be well-contested?*

First, regarding the timing of SIA campaigns, we assumed that all vaccination (both RCV2 and SIAs) occurred at the beginning of each year to ensure that cohorts are being vaccinated with the proper coverage. By doing this, we ensure that the proportion of susceptibles relative to recovered remains adequate for each cohort, to then model disease dynamics for the remainder of the year. As results are reported on a yearly basis, intra-annual variations do not affect results.

Second, regarding the cases introduced, as the model is driven by both the number of infected cases in the population and the average force of infection, it could be the case that the disease was eradicated only because a significant outbreak occurred in a short period of time. Without imported cases, the proportion of infected would remain below a threshold that would prevent additional cases for the rest of the study period. The introduction of new cases each year was done to avoid this 'artificial' eradication of rubella. Additionally, as far as we know there is no quality data regarding the imported cases in the studied countries, and therefore, we assumed a constant number of cases each year based on empirical experiments that we conducted.

4. *Additionally, we assumed that during the first year of introduction the RCV coverage would correspond to 50% of the MCV immunization coverage and kept constant at 100% for the following years of the planning horizon for each country. This is overly ambitious and unrealistic, given the immunization trends/history in these countries of interest (NGA, PAK, COD etc.).*

We believe that we were not clear enough about our statement. We do assume that RCV coverage will match 50% of the current MCV coverage the first year, and 100% of it in the following years. Thus, if a country has MCV1 and MCV2 coverage of 80% and 30% respectively, then for the first year of introduction RCV1 and RCV2 coverage will correspond to 40% and 15%. For the following years, RCV1 and RCV2 coverage will be 80% and 30% respectively. As we assume that the introduction will be through the replacement of only-measles containing vaccines by combination vaccines, we believe that is realistic to assume that future coverage will be close to the current MCV coverage. We clarified our statement in Section 2.2 (see lines 125-128).

5. *We also estimated the number of CRS births by considering the three varying levels of projected 159 births (low (L), medium (M) and high (H)) from (27)? Guess there is a typo here.*

We thank the reviewer for pointing this out, we have corrected the typo in the manuscript.

6. *In the method section, what guided the assumptions made on the set points for the follow-up in the various scenarios, particularly scenarios 3, 4 and 5. For completeness, the author may want to state this.*

The scenarios were designed based on those proposed in Motaze et al. 2020. The rationale for follow-up SIAs timing is that they target children in the 0-4 years old age group, hence, a new cohort is vaccinated every 5 years. We included this clarification in Section 2.2.1 (see lines 153-155).

7. *Regarding the limitation, could the validity and reliability (quality) of the underlying routine immunization data impact the outcome of the modeling study for the selected countries?*

We agree with the reviewer that this is an important limitation to discuss, and we have added this to the Discussion section (see lines 343-346). To the best of our knowledge this is the best source as of today for immunization coverage[2].

[2] Rau C, Lüdecke D, Dumolard LB, et al. Data quality of reported child immunization coverage in 194 countries between 2000 and 2019. PLOS Glob Public Health. 2022;2(2):e0000140. Published 2022 Feb 3. doi:10.1371/journal.pgph.0000140
